# Effects of dance therapy on self-efficacy and mental health of college students

**DOI:** 10.3389/fpsyg.2026.1837431

**Published:** 2026-07-09

**Authors:** Hongmei Li, Di Wu

**Affiliations:** 1 School of Music and Dance, Harbin University, Harbin, Heilongjiang, China; 2 College of Sports and Art, Harbin Sport University, Harbin, Heilongjiang, China

**Keywords:** college students, dance therapy, effect of dance therapy, mental health, self-efficacy

## Abstract

Cultivating mental health talents is one of the most important goals of education, and some college students have varying degrees of mental health problems. Meanwhile, self-efficacy is an important concept in social cognitive theory and has significant health functions. This paper attempts to investigate the impact of dance therapy on the self-efficacy and mental health of college students. This paper used the SCL-90 Symptom Checklist to evaluate 462 college students twice before and after 3 months of dance exercise, and compared the results before and after the experiment. The experiment found that after 3 months of dance therapy, the average self-efficacy score of boys was 27.85 points, higher than that of girls at 26.84 points. All other scores were lower than the test scores before the experiment, indicating that the mental health of college students has improved after receiving dance therapy.

## 1 Introduction

The general decline in the physical fitness of our college students in recent years has also had a knock-on effect on their mental health, to the point that colleges are now filled with highly educated but undertrained individuals with high IQs and low emotional abilities. High education and high IQ are the result of the accumulation of conceptual knowledge, while low education and low EQ are mainly due to inability to deal with interpersonal relationships, poor self-management ability, and inability to adjust their thinking in the face of failures and setbacks. Strict courses and severe work situation have increased the psychological pressure of college students. College students who grew up in the unique way of “only paying attention to learning cultural knowledge” cannot bear this psychological pressure. College students are a special group, their thoughts gradually mature at this time, and their mental health affects the formation of their outlook on life, values and world outlook. Personal and emotional problems, study and life problems, and employment problems after graduation make their psychological burden even heavier. It is becoming more and more urgent to find ways to relieve this mental stress and improve mental health.

Fitness and mental health are shaped by social determinants such as ethnicity, race, class, and gender. Hyun’s ethnography explored how low socioeconomic status (SES) affects college students’ mental health and self-efficacy. Through literature review and interviews at the State University of New York at Geneseo, he found that students’ mental health and self-efficacy are strongly linked to low SES (Haerreem, 2018). Ersoz examined the relationship between exercise, self-efficacy, depression, and mental health in 522 college students using the General Self-Efficacy Scale (GSES) and Beck Depression Inventory (BDI). ANOVA and Pearson correlation analysis revealed that higher exercise levels corresponded with greater self-efficacy, better mental health, and lower depression (Ersoz, 2017). Chau studied 520 students in Lima, employing the SF-36, GSE, PSS, and COPE-60. Results indicated men had higher physical fitness, students with fewer academic difficulties had better psychological wellbeing, and higher perceived stress was linked to lower physical and mental health (Chau and Vilela, 2017a). Cho investigated 228 out-of-school adolescents, finding that higher self-efficacy reduced vulnerability to mental health issues, and family support moderated this relationship, highlighting the importance of social support systems (Hong, 2017). Wilcox studied 66 incoming freshmen, identifying that academic satisfaction and school connectedness predicted life satisfaction, while scholastic self-efficacy and college wellbeing predicted academic performance (Wilcox and Nordstokke, 2019). Overall, these studies underscore the importance of social, behavioral, and psychological factors in shaping students’ mental health and self-efficacy, suggesting interventions that promote exercise, social support, and school connectedness can enhance college students’ wellbeing.

Dance therapy is the utilization of dance or improvisation to treat social, emotional, cultural and physical impairments, as well as to heighten personal consciousness and enhance people’s thinking. Wiedenhofer S explored the effects of non-goal directed improvised dance versus goal directed improvised dance in reducing perceived stress, improving wellbeing, general self-efficacy and physical self-efficacy. Participants in the experimental group (EG) performed non-goal-oriented improvised dance movements, whereas participants in the control group (CG) improvised the same music in a goal-oriented manner with the help of colored pieces of paper as targets. The results showed that non-goal-oriented improvised dance and goal-oriented improvised dance were effective in increasing physical self-efficacy and superior to goal-oriented improvised dance in reducing perceived stress. In addition, improvised dance in general appears to have beneficial effects on health-related psychological outcomes. Future research should investigate the impact on clinical settings, identify other positive factors of dance therapy, and address it theoretically (Wiedenhofer and Koch, 2017). The therapeutic motor relation (TMR) is core to the practice of life as a distance practitioner in dance/movement therapy; however, a definition of this core concept is still lacking. Young explored the meaning and nature of TMR as a result of a joint study conducted by eight accredited dance/movement therapists to reach a definition. Data collection from semi-structured surveys yielded five textual themes that describe the nature of TMR: these informed the development of a diagnosis of TMR that can sustain the practice, study, supervision, and reform of this important part of dance/movement therapy (Jessica, 2017). These methods have provided some data for our study, but have not yet gained public recognition due to the short duration z and small sample size of the relevant studies.

Against the above research background, existing studies on dance therapy and college students’ mental health are mostly limited to small samples, short intervention cycles, and single-dimensional outcome indicators, lacking systematic exploration of the nonlinear relationships between self-efficacy and mental health. Moreover, few studies have integrated advanced statistical modeling with psychological intervention evaluation, resulting in insufficient empirical evidence for the mechanism and effectiveness of dance therapy. To address these gaps, this study adopts a rigorous randomized controlled trial design, combining standardized psychological scales with Copula entropy and weighted naive Bayesian modeling, to systematically examine the intervention effects of dance therapy on college students’ self-efficacy and mental health. It further clarifies the differential impacts of demographic characteristics on intervention outcomes, aiming to enrich the theoretical system of dance therapy in mental health promotion, provide reliable empirical support for the popularization of mind–body interventions in college mental health services, and offer practical references for developing targeted psychological intervention strategies for college students.

## 2 Methods

### 2.1 Dance therapy

The concept of dance therapy originated in Europe at the end of the 19th century and first appeared in the United States. The British Dance Therapy Association defines it as “people’s creative engagement in the therapeutic process to improve their emotional, cognitive, physical and social integration through the application of scientific therapeutic principles to the stage of dance movement (Chau and Vilela, 2017b). Dance therapy is very flexible, interactive and creative. It enables practitioners to fully understand their own personality, emotions and thinking, and to become aware of their own community and environment, thereby achieving a state of stability and improved physical and mental health. In the process of development of dance therapy, the system has been gradually improved, the professionalization and scientific level have become increasingly enhanced, and its influence has been continuously expanded on a global scale (Berte et al., 2019). Although China started late in introducing dance therapy, dance therapy, as a relatively young and promising discipline, has been recognized and applied in various fields such as art, education, and medical care.

Dance therapy begins with a preparation phase. The preparation phase, that is, the mental and physical warm-up, helps the experimenter relieve psychological anxiety and establish a sense of security, so that he can participate in therapeutic activities in a more relaxed mental state. The educational methods used help to improve students’ self-awareness and sense of identity (Christina, 2017).

In today’s society, under the influence of the Internet and fragmented information, while people enjoy the convenience and speed brought by the Internet, the direct emotional communication between each other is gradually replaced by virtual interaction, which undoubtedly makes young students become direct victims. In dance therapy classes, teachers provide enough space for college students to release their bodies and use their imaginations (Li, 2021). The physical display performed by students in an unconscious state is the most realistic portrayal of their hearts, and it is also the most direct release of the pressures and difficulties they face in real life. The meditation method can relax the body and mind of the participants, thereby indirectly stimulating the creative enthusiasm and innovation ability of the students’ subconscious mind (Yefei and Zhang, 2017).

### 2.2 Self-efficacy

**Figure 1:**
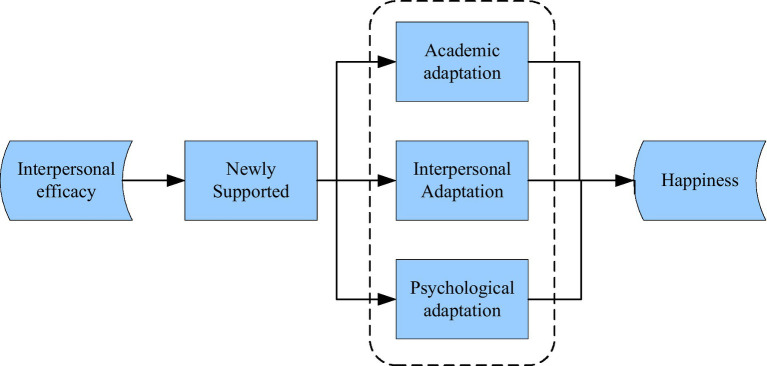
The relationship between self-efficacy and wellbeing.

Self-efficacy is a subjective factor that affects a person’s choice and content of activities. It is not a person’s behavior per se, but the mediating factor between a person’s motivation and his behavior. It has the characteristics of behavioral motivation (Green et al., 2021). Therefore, self-efficacy plays an important role in an individual’s behavior. People with high self-efficacy received more social support in the new environment. The more social support a person receives, the faster and better he or she adapts to the new environment. Social support is most closely related to social adaptation, followed by psychological and academic adaptation (Kholmogorova et al., 2019). The higher the level of adaptation, the higher the individual’s wellbeing. The relationship between self-efficacy and wellbeing is shown in Figure 1.

This study assessed and analyzed college students’ self-efficacy in strict accordance with psychometric norms, employing standardized scales, rigorous administration procedures, and quantitative statistical models to systematically capture the dynamic changes in self-efficacy before and after dance therapy intervention, providing objective and reliable data support for evaluating the intervention effect (Lau et al., 2018; Mcmeel et al., 2017). Self-efficacy, as an individual’s subjective belief in their ability to complete specific tasks, is a core psychological variable for predicting mental health and behavioral adaptation. Its accurate assessment is crucial for revealing the mechanism of action of dance therapy (Bashir, 2018).

The study used the GSES as the assessment tool. The scale contains 10 items, using a 4-point Likert scoring method, ranging from “1 = completely disagree” to “4 = completely agree.” Subjects were required to answer based on their true feelings, with no reverse-scoring items. The total score ranged from 10 to 40 points, with higher total scores indicating stronger self-efficacy.

The administration process strictly followed standardized procedures, conducted intensively one day before the intervention (baseline) and the day after the intervention (final test). Before administration, trained examiners read out the instructions, clearly explaining the purpose of the assessment, completion requirements, confidentiality principles, and time allotted for answering. Emphasis was placed on independent and truthful responses to alleviate participants’ concerns and ensure the quality of their answers. The assessment was conducted collectively by class, with questionnaires collected on-site. Invalid questionnaires, including those with missed answers, incorrect answers, or patterned responses, were immediately discarded to ensure data validity. To further control systemic errors, the assessment was conducted in a quiet, well-lit, and undisturbed classroom, with the answering time limited to 10 min to avoid fatigue affecting the accuracy of the results.

Data entry and preprocessing employed a dual-person, dual-entry method. An Excel database was used to cross-check the entered data, correcting errors and ensuring 100% accuracy. The data was then imported into SPSS 26.0 software for statistical analysis. First, the reliability of the scale was tested, and internal consistency was assessed using Cronbach’s *α*. The calculation equation is as follows:

$$\alpha =\frac{k}{k-1}(1-\frac{\sum_{i=1}^{k}\sigma_{i}^{2}}{\sigma_{X}^{2}})$$ (1)

In the Equation 1, k is the number of items in the scale, $\sigma_{i}^{2}$ is the variance of the score of the *i*-th item, and $\sigma_{X}^{2}$ is the variance of the total score of the scale.

In terms of statistical analysis, the total self-efficacy scores and mean scores of each item in the experimental and control groups before and after intervention were first calculated, and the data distribution characteristics were presented through descriptive statistics. Then, an independent samples *t*-test was used to test the baseline balance between the groups, confirming that there was no significant difference in self-efficacy levels between the two groups before intervention. Finally, repeated measures ANOVA was used to test the interaction effect between groups and time, quantify the intervention effect of dance therapy on self-efficacy, and the effect size was represented by Cohen’s d value to clarify the effect size and ensure the scientific validity and persuasiveness of the statistical conclusions.

### 2.3 Importance of mental health of college students

Mental health is a complex and multidimensional concept that encompasses not only modern psychology. It also includes sociological, medical and other aspects, which is itself an interdisciplinary study (Honkala, 2019). Generally speaking, it consists of two main aspects: physical health and mental health. In a narrow sense, mental health refers to the absence of physical and mental illness, that is, the body functions normally and there are no diseases that significantly affect the body. In a broader sense, mental health refers to a person’s adjustment to his or her environment. Adaptation refers to coping with an optimistic and positive mental attitude, while being aware of one’s current situation. By understanding their current emotional state, they become aware of their external needs and psychological development level (Schwindt et al., 2019). Through training and practice, people can improve their mental state and adapt to changes in the external environment in the best possible way. It effectively develops one’s physical and mental potential and positive social functioning (Lippi and Petit, 2017).

Different definitions of mental health suggest that it evolves over time and across different social contexts, but the common thread is the ability to cope and enjoy life in its social environment. College students’ mental health means that college students should not only have normal intelligence, but also have a positive and optimistic attitude towards life. It is willing to communicate with people, establish harmonious interpersonal relationships, perseverance and self-improvement in the face of difficulties. It treats others fairly and consciously, and has good character and psychological quality. The mental health of college students is not only closely related to their study life, but also affects their future growth and development. It is actually vital to their lifelong development. Mental health is also an important prerequisite for students to gain academic knowledge and lead a successful academic life. Only mental health can enable students to actively participate in academic activities. It prepares for difficulties and problems, considers them carefully, and actively seeks solutions and strategies. Only mental health can enable college students to establish good interpersonal relationships, actively interact with the social environment, and obtain more social resources and social support. Mental health is also a prerequisite for students’ successful integration into society and the labor market. Mental health is the cornerstone and foundation of a student’s career development, no matter what kind of job they will have in the future. In today’s increasingly competitive and fast-moving social environment, work will undoubtedly be difficult and demanding. So in this new, more challenging environment, maintaining mental health and personal independence is especially important for work and career development.

Students’ mental health is closely related to their studies, social life, daily life and work, and it is a necessary condition for them to easily integrate into university life and live fully. Whether college students can adapt well when they enter the university plays an important role in determining their mental health in the future.

### 2.4 Correlation between dance therapy and college students’ self-efficacy and mental health

With the development of psychology, more and more studies have shown that mental and physical health are closely linked and complement each other. On the one hand, many clinical physical diseases are often accompanied by psychological problems. On the other hand, psychological problems can also lead to physical illnesses. For example, chronically anxious people are more sensitive to external stimuli that trigger a wide range of physiological responses. It has a large effect on the hyperactivity of the autonomic nervous system, which becomes exhausted when stimulated for a long time without rest. This can lead to various diseases. In addition, good psychological quality has a better impact on the treatment and prognosis of physical diseases. The above facts show that mind and body are not two opposing aspects, but are closely intertwined, and it is the interaction between body and environment that creates mind and cognition. Thus, the effects of physical and mental health are reciprocal. Based on this, the concept of Copula entropy was proposed as a measure of the correlation between dance therapy and college students’ self-efficacy and mental health. It is used to assess the impact of various factors.

The earliest concept of Copula appeared in probability and statistics theory. Copula functions are essentially a general term for a class of functions that relate joint functions to their respective marginal distributions. The basic principle of Copula can be boiled down to the following Equation 2:

$$F(x_{1},x_{2},\ldots ,x_{N})=C(F_{1}(x_{1}),F_{2}(x_{2}),\ldots ,F_{N}(x_{N}))$$ (2)

$F(x_{1},x_{2},\ldots ,x_{N})$ represents the joint distribution function required in the model, C represents the Copula function structure, and $F_{1}(x_{1}),F_{2}(x_{2}),\ldots ,F_{N}(x_{N})$ represents the respective marginal distributions of multiple variables. If $F_{1}(x_{1}),F_{2}(x_{2}),\ldots ,F_{N}(x_{N})$ is continuous, C is uniquely determined. On the contrary, in discrete data experiments, C can be estimated by other means.

Copula functions of a joint distribution can be written as the inverse of their marginal functions and the combination of the joint functions. Another basic common form of the above model is Equation 3:

$$C(\omega_{1},\omega_{2},\ldots ,\omega_{N})=F(F_{1}^{\left(-1\right)}(x_{1}),F_{2}^{\left(-1\right)}(x_{2}),\ldots ,F_{N}^{\left(-1\right)}(x_{N}))$$ (3)

Through the density function c of the Copula function, it is easy to know the relationship between the joint distribution function of the Copula function, the density function and the marginal density, as Equation 4:

$$f(x_{1},x_{2},\ldots ,x_{N})=c(\begin{array}{l} F_{1}(x_{1}),F_{2}(x_{2}),\ldots ,\\ F_{N}(x_{N}) \end{array})\prod_{n=1}^{N}f_{n}(x_{n})$$ (4)

Among them

$$c(\omega_{1},\omega_{2},\ldots ,\omega_{N})=\frac{\partial C(\omega_{1},\omega_{2},\ldots ,\omega_{N})}{\partial \omega_{1}\partial \omega_{2}\ldots \partial \omega_{N}}$$ (5)

$f_{n}(\cdot ),n=1,2,\ldots ,N$ is the density function of the marginal distribution function $F_{n}(\cdot ),n=1,2,\ldots ,N$ .

According to the Equation 5, Copula entropy $H_{c}(\omega_{1},\omega_{2},\ldots ,\omega_{n})$ is defined as the following equation:

$$\begin{array}{l} H_{c}(\omega_{1},\omega_{2},\ldots ,\omega_{n})=\int_{-\infty }^{+\infty }\ldots \int_{-\infty }^{+\infty }c(\omega_{1},\omega_{2},\ldots ,\omega_{n})\\ \mathrm{lnc}(\omega_{1},\omega_{2},\ldots ,\omega_{n})d\omega_{1},\ldots ,d\omega_{n} \end{array}$$ (6)

In the Equation 6, $\left(\omega_{1},\omega_{2},\ldots ,\omega_{n}\right)$ is the density function corresponding to the n-dimensional Copula function C, and its corresponding marginal distribution function is expressed as the Equation 7:

$$\omega_{i}=F_{i}(X_{i})=P(x_{i}\leq X_{i}),i=1,2,\ldots ,n$$ (7)

Equation 5 can also be in vector form, as the Equation 8:

$$H_{c}(W)=\int_{+\infty }^{-\infty }c(W)\mathrm{lnc}(W)\mathrm{dW}=E[\ln\;c(W)]$$ (8)

According to the definition of discrete Copula, Copula entropy can be transformed into the Equation 9:

$$E[\mathrm{lnc}(W)]=\sum_{W=1}^{n}c(\frac{W}{n})\mathrm{lnc}(\frac{W}{n})$$ (9)

Compared with the concept of entropy, the unit of Copula entropy function $H_{c}$ is still nat.

$$H(x_{1},x_{2},\ldots ,x_{n})=\sum_{i=1}^{n}H(x_{i})+H_{c}(\omega_{1},\omega_{2},\ldots ,\omega_{n})$$ (10)

According to the Equation 10, the Copula entropy is decomposed from the joint entropy number $H_{c}(\omega_{1},\ldots ,\omega_{i},\ldots ,\omega_{n})$ , and the Copula entropy structure is used as the correlation index in the variable structure.

**Figure 2:**
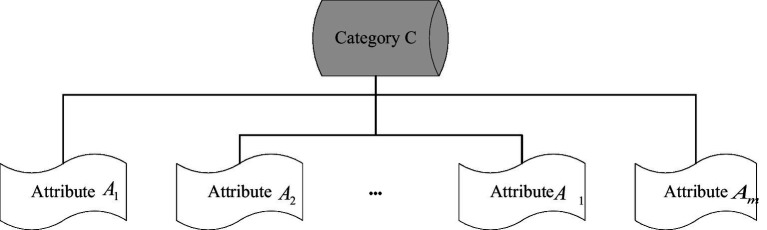
Classifier structure based on Naive Bayes algorithm.

The structure diagram of the classifier based on the Naive Bayes algorithm is shown in Figure 2.

In the figure, the leaf node represents the *m*th attribute, and the root node *C* represents the category. Let D={C,A,S} be a training sample, which includes student category $C=\{C_{1},C_{2},\ldots ,C_{i}\}$ and student attribute $A=\{A_{1},A_{2},\ldots ,A_{m}\}.$

**Figure 3:**
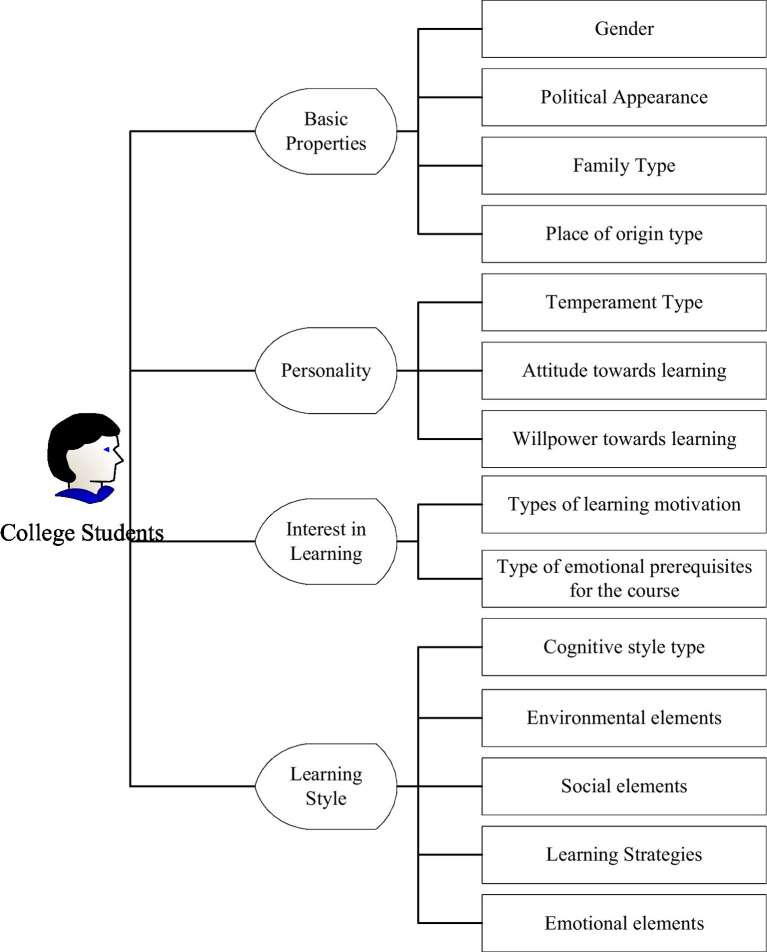
Personalization attributes of students.

A total of 14 attributes in 4 dimensions are selected to describe a student’s individuality, as shown in Figure 3.

According to the Equations 11, 12, let the sample space of experiment *S* be *T*, *L* be the events of S, $M_{1},M_{2},\ldots ,M_{n}$ be a division of T, and

P(L)>0,P(M)>0(i=1,2,…,n) (11)

Then

$$P(M_{i}|L)=\frac{P(L|M_{i})P(M_{i})}{\sum_{j=1}^{n}P(L|M_{j})P(M_{j})},i=1,2,\ldots ,n$$ (12)

Let R be the training set, and use $D_{1},\ldots ,D_{n},E$ to denote n attribute variables and class variables, respectively. $E_{1},E_{2},\ldots ,E_{n}$ is m known categories, and the class variable set is expressed as the Equation 13:

$$E_{1},E_{2},\ldots ,E_{n}$$ (13)

The attribute variable set is represented as the Equation 14:

$$D=\{D_{1},\ldots ,D_{j},\ldots ,D_{n}\}$$ (14)

And $\left\{d_{1},\ldots ,d_{j},\ldots ,d_{n},c_{i}\right\}$ is used to represent a single training sample, $\left\{d_{1},\ldots ,d_{j},\ldots ,d_{n}\right\}$ is used to represent the test sample X, and $d_{1},\ldots ,d_{j},\ldots ,d_{n}$ represents the value of the attribute variable $D_{1},\ldots ,D_{j},\ldots ,D_{n}$ respectively. The probability that the test sample X belongs to class $E_{i}(i\in [1,m])$ is as Equations 15, 16:

$$P(E_{i}|d_{1},d_{2},K,d_{n})=\frac{P(d_{1},d_{2},K,d_{n}|E_{i})\cdot P(E_{i})}{P(d_{1},d_{2},K,d_{n})}$$ (15)

$$P(d_{1},d_{2},K,d_{n}|E_{i})=\prod_{j=1}^{n}P(d_{j}|E_{i})$$ (16)

For a sample to be classified

$$X=\{d_{1},\ldots ,d_{i},\ldots ,d_{n}\}$$ (17)

According to the Equation 17, when the naive Bayes classification algorithm determines that the posterior probability $P(E_{i}|d_{1},d_{2},\ldots ,d_{n})$ takes the maximum value, the corresponding category is used as the class label of the sample to be classified, that is, the classification result is as the Equation 18:

$$\arg\max_{E_{i},i\in [1,m]}P(E_{i})\prod_{j=1}^{n}P(d_{j}|E_{i})$$ (18)

In this paper, a weighted naive Bayesian classification algorithm based on Tau-y coefficient is proposed. The weight $\omega_{t}$ is the Tau-y coefficient. The weighted equation is as the Equation 19:

$$P(X|E_{i})=\omega_{t}\cdot \prod_{j=1}^{n}P(x_{j}|E_{i})$$ (19)

In the above equation, $\omega_{t}$ is the weight. The classification result is as the Equation 20:

$$\arg\max_{E_{i},i\in [1,m]}P(E_{i})\prod_{j=1}^{n}P{\left(d_{j}|E_{i}\right)}^{\omega_{t}}$$ (20)

### 2.5 Data source

#### 2.5.1 Objects

**Figure 4:**
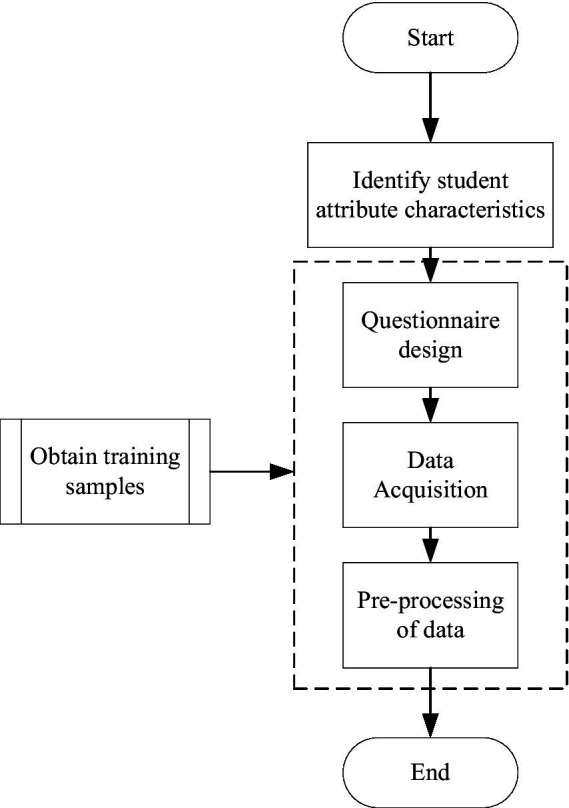
Flowchart of the preparation phase.

Based on Naive Bayesian algorithm and probability equation, this paper proposes a student classification method based on Naive Bayesian algorithm. The whole process of classification method is roughly divided into three stages: preparation, training and application. The research flow chart of the preparation stage is shown in Figure 4.

This study employed a randomized controlled trial design. All participants signed informed consent forms, clearly understanding the research objectives, intervention procedures, potential risks, and their right to voluntarily withdraw. The entire process adhered to the principles of voluntariness, anonymity, and confidentiality. Participants were randomly assigned to groups in a 1:1 ratio. A pre- and post-test parallel controlled design was used, employing stratified random sampling based on academic disciplines. 500 questionnaires were randomly selected from university students, and 489 were returned, representing a response rate of 97.8%. After eliminating invalid questions, there were 462 effective copies of questions, with an efficiency ratio of 94.48%. To evaluate the effect of dance therapy on college students’ mental health, this study was divided into experimental and control groups according to whether they participated in dance therapy or not.

The control group consisted of 206 valid participants, all of whom provided informed consent prior to enrollment. These students took part in a standardized 3-month dance therapy intervention totaling 90 days, with four sessions scheduled every other day each week, and each session lasting 2 h. Every session adhered to a rigorous three-phase structure aligned with established dance/movement therapy guidelines. The warm-up phase lasted 20 min, incorporating gentle rhythmic movements, breath regulation, and somatic awareness exercises to ease psychological anxiety and establish a secure therapeutic environment. The core intervention phase took 90 min, including structured dance improvisation, expressive movement practice, group dance routines, and non-verbal emotional expression tasks aimed at strengthening body awareness, facilitating emotional release, and improving interpersonal communication. The final 10-min cool-down and reflection phase involved slow stretching, guided mindfulness practice, and short group sharing to help participants integrate their inner experiences and maintain physical and mental relaxation.

The basic situation scale of college students is investigated and analyzed from the aspects of gender, major, place of origin, whether they are only children, and family economic situation. The results are shown in Tables 1, 2.

**Table 1:** Survey on the basic situation of college students.

Test Items		Number of people	Proportion
Gender	Male	262	56.71%
	Female	200	43.29%
Specialties	Science	89	19.26%
	Humanities	121	26.19%
	Engineering	91	19.70%
	Agricultural Science	100	21.65%
	Medical Science	61	13.20%

**Table 2:** Detailed personal information survey form for college students.

Test items		Number of people	Proportion
Only child	Yes	275	59.52%
	No	187	40.48%
Monthly living expenses	<500	85	18.40%
	≥500	377	81.60%
Source of students	Rural	223	48.27%
	Cities and towns	239	51.73%

#### 2.5.2 Psychological test scales

This paper uses the SCL-90 test to assess the mental health of college students in terms of mood, emotion, thinking, behavior, habits, interpersonal relationships, diet, and sleep. And it compares the scores before and after the test. The SCL-90 is a 90-item scale. It has 10 indirect factors (somatization, compulsion, interpersonal sensitivity, depression, anxiety, hostility, fear, delirium, psychosis, etc.). It was adapted from foreign scales by Chinese psychologists. The results showed that the semi-reliability of the 10 subscales was between 0.565 and 0.850, and the Cronbach’s coefficient was between 0.754 and 0.862. This indicates this measure holds excellent credibility in the college student population. The collinearity among the 10 subscales and the sum score varied from 0.705 to 0.9, and the cultivation among the scales ranged from 0.49 to 0.824. The findings suggest that the measure possesses excellent overall validity and structural stability.

A total SCL-90 score of ≥70 was used as a positivistic test for the detection of behavioral health issues in this paper. The outcome is shown in Table 3.

**Table 3:** Number and proportion of SCL-90 positive symptoms.

Total score of the symptom self-assessment scale	Number of people	Proportion
≥70	101	21.86%
<70	361	78.14%
Total	462	100.00%

## 3 Results

### 3.1 Influence of dance therapy on the self-efficacy of college students

**Figure 5:**
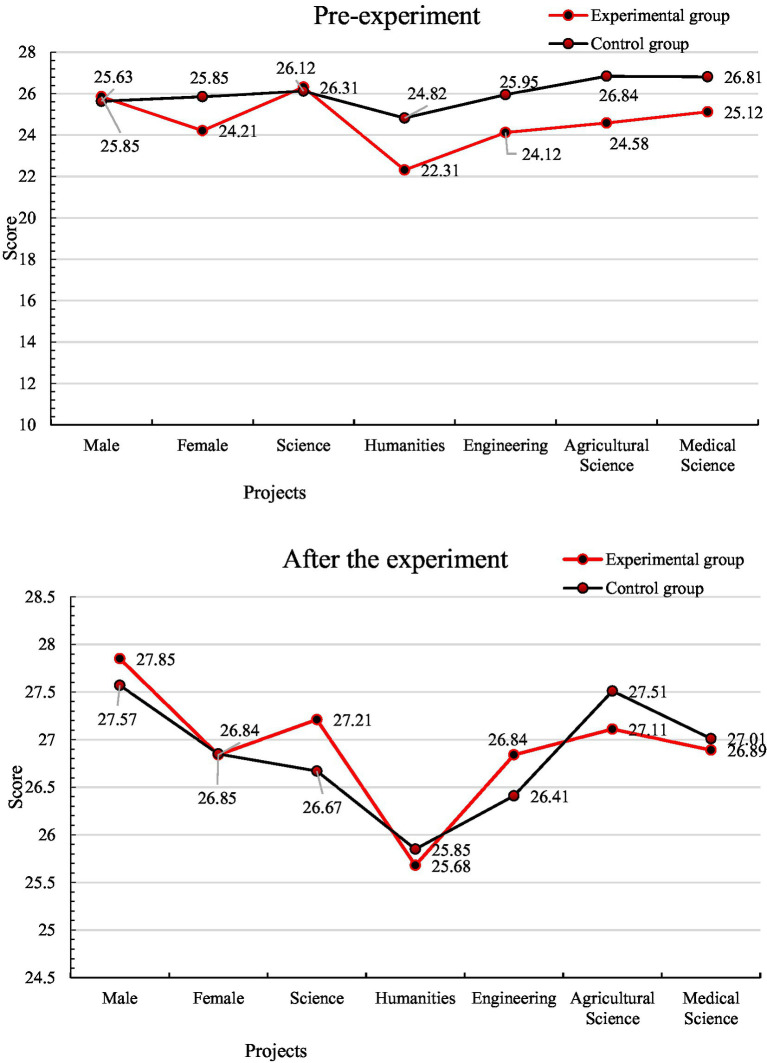
Differences in self-efficacy before and after the experiment among college students of different genders and majors.

**Figure 6:**
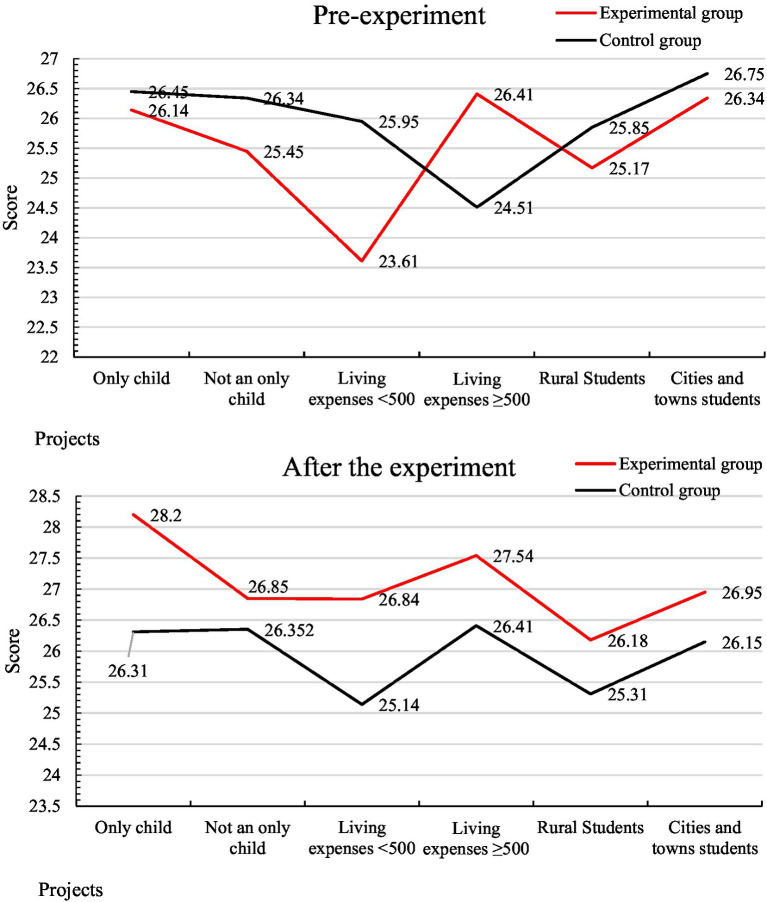
Differences in self-efficacy of college students with different family backgrounds before and after the experiment.

Self-efficacy manifests in different forms in different fields, including general self-efficacy, subject-specific self-efficacy, and interpersonal self-efficacy. This study only measures and analyzes general self-efficacy. Figures 5, 6 are the analysis of the influence of dance therapy on the self-efficacy of college students with different basic conditions.

From Figures 5, 6, it can be seen that the self-efficacy scores of college students of different genders have been greatly improved after 3 months of dance therapy. The average score of boys is 27.85, which is higher than the average score of girls 26.84. After analyzing and comparing the differences in the total self-efficacy scale of college students of different majors, it was found that the self-efficacy total scale of college students of different majors after dance therapy was quite different, and the improvement value reached 3.37. The lowest is only 0.9, but after the experiment, students of all majors generally show an upward trend in their self-efficacy scores.

### 3.2 Influence of dance therapy on the mental health of college students

**Figure 7:**
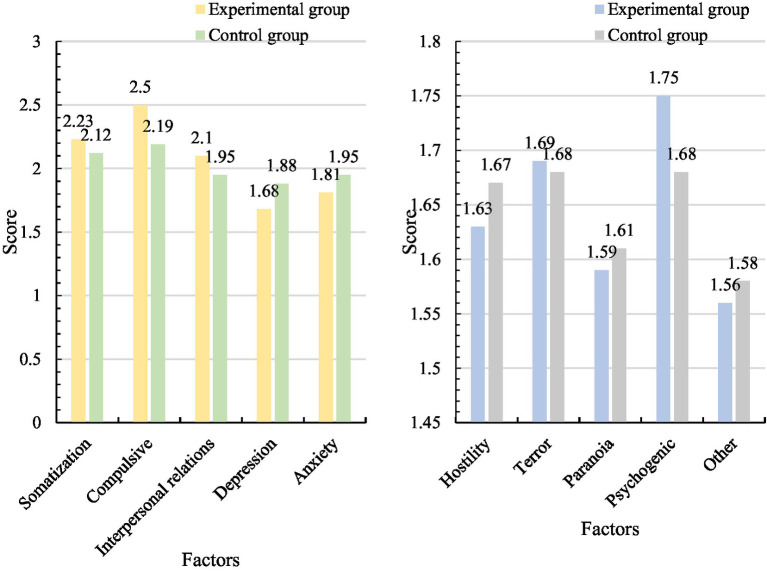
Comparative analysis of the mental health of the two groups of subjects before the experiment.

Figure 7 is a comparative analysis of the mental health of the two groups of subjects before the experiment. The 10 factors of mental health of the students in the two groups were tested, and the experimental group was significantly different from the control group in the two factors of obsessive-compulsive symptoms and psychosis. The former score was significantly higher than that of the control group, with a difference of 0.31. The latter was significantly lower than the control group, with a difference of 0.07. The rest of the factors indicated that there were no significant differences.

**Figure 8:**
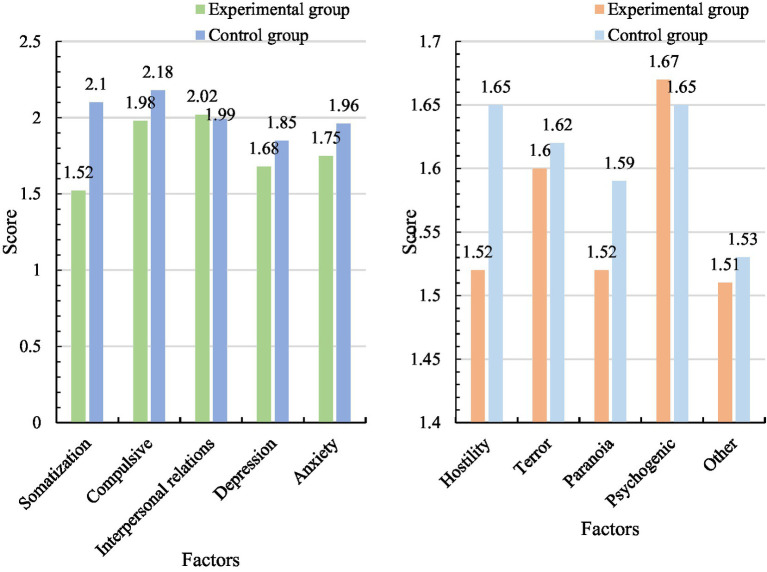
Comparative analysis of the mental health of the two groups of subjects after the experiment.

Figure 8 is a comparative analysis of the mental health of the two groups of subjects after the experiment. It can be seen from the figure that after 3 months of teaching practice, the dimension levels of the students in the experimental group are basically the same as before the experiment except for the depressive symptom factors. The rest of the scores are lower than the test results before the experiment. It formed a more obvious contrast with the control group, in which the factor of somatic symptomatization had the most obvious change, which decreased from 2.23 to 1.52. It shows that the mental health level of the tested college students who have undergone dance therapy has been improved.

To further quantify the effect of dance therapy on the mental health of college students, this study used paired samples *t*-test and repeated measures ANOVA to perform inferential statistical analysis on the pre-test and post-test scores of each factor of the SCL 90 in the experimental group, and clarified the significance of differences, *t*-values, *p*-values and effect sizes. The results are shown in Table 4.

**Table 4:** Pre- and post-test inference statistics of SCL-90 factors in the experimental group.

SCL-90 factor	Pre (M ± SD)	Post (M ± SD)	*t*	*p*	Cohen’s *d*	Effect level
Somatization	2.23 ± 0.41	1.52 ± 0.35	11.35	<0.001	0.91	Large
Obsessive-compulsive	2.50 ± 0.48	1.98 ± 0.42	8.72	<0.001	0.72	Large
Interpersonal sensitivity	2.10 ± 0.39	2.02 ± 0.37	1.86	0.064	0.21	Small
Depression	1.68 ± 0.34	1.68 ± 0.33	0	1	0	Negligible
Anxiety	1.81 ± 0.40	1.75 ± 0.36	3.21	0.002	0.26	Small
Hostility	1.63 ± 0.32	1.52 ± 0.29	4.15	<0.001	0.36	Medium
Phobic anxiety	1.69 ± 0.35	1.60 ± 0.31	3.08	0.002	0.27	Small
Paranoid ideation	1.59 ± 0.30	1.52 ± 0.28	2.47	0.014	0.24	Small
Psychoticism	1.75 ± 0.34	1.67 ± 0.31	2.51	0.013	0.25	Small
Other	1.56 ± 0.29	1.51 ± 0.27	1.89	0.06	0.18	Small

Statistical results showed that after 3 months of dance therapy intervention, the experimental group showed significantly lower post-test scores than pre-test scores on somatization, obsessive-compulsive, anxiety, hostility, phobia, paranoia, and psychoticism factors (*p* < 0.05). The somatization factor showed the most significant improvement (*t* = 11.35, *p* < 0.001), with an effect size of d = 0.91, reaching a large effect level; the obsessive-compulsive factor also showed significant improvement (*t* = 8.72, *p* < 0.001), with an effect size of *d* = 0.72, also a large effect. The hostility factor showed an effect size of d = 0.36, a moderate effect; the other significantly improved factors all had small effects. Repeated measures ANOVA showed a significant between-group × time interaction (*F* = 28.65, *p* < 0.001), indicating that the intervention effect was not due to time or natural maturation. There was no significant difference in the pre- and post-test scores for the depression factor (*t* = 0.00, *p* = 1.000).

### 3.3 Statistical and model performance analysis

To verify the statistical reliability of the research conclusions and quantify the predictive ability of the model, this paper introduces inferential statistical tests, effect size analysis, and a Copula entropy-Naive Bayes fusion model to systematically evaluate the nonlinear association between the intervention effect of dance therapy and psychological indicators. The significance level for all statistical tests was set at 0.05. Model performance was comprehensively evaluated using four core indicators: classification accuracy, precision, F1 score, and AUC-ROC. The results are shown in Table 5.

**Table 5:** Inferential statistical results of differences in self-efficacy before and after intervention in the two groups.

Comparison dimensions	Group	Mean ± standard deviation	*t*	*p*	Cohen’s *d*	Effect level
GSES total score before intervention	Experimental group	25.18 ± 3.42	1.24	0.216	0.08	Negligible
	Control group	25.09 ± 3.45				
GSES total score after intervention	Experimental group	27.32 ± 3.15	12.76	<0.001	0.65	Medium
	Control group	25.21 ± 3.38				
Before and after comparison of the experimental group	Before intervention vs. after intervention	–	12.76	<0.001	0.65	Medium
Before and after comparison of the control group	Before intervention vs. after intervention	–	1.02	0.308	0.06	Negligible

Table 5 quantifies the differences in self-efficacy between and within groups, validating the intervention’s effectiveness from both inferential statistics and effect size dimensions. At baseline, there was no statistically significant difference in the total GSES scores between the two groups (*t* = 1.24, *p* = 0.216), Cohen’s *d* = 0.08, confirming baseline comparability and excluding the interference of initial differences. After the intervention, the experimental group showed a significant improvement in self-efficacy, with a highly significant difference between groups (*t* = 12.76, *p* < 0.001), and an effect size of 0.65 (moderate effect), indicating that dance therapy has substantial clinical significance in improving self-efficacy. The control group showed no significant change (*t* = 1.02, *p* = 0.308), with an effect size of only 0.06, further excluding time or natural maturation effects.

To verify the practicality of the Copula entropy and Naive Bayes fusion model in predicting the effects of psychological interventions, Table 6 presents the core performance indicators of the model in the binary classification task of “whether mental health has improved or not.”

**Table 6:** Copula entropy–Naive Bayes model classification performance metrics.

Evaluation metrics	Numerical values	95% confidence interval	Performance rating
Accuracy	0.862	[0.825, 0.891]	Good
Precision	0.847	[0.806, 0.879]	Good
F1-score	0.854	[0.815, 0.885]	Good
AUC-ROC	0.875	[0.838, 0.902]	Good

The model uses dance therapy intervention parameters and baseline psychological indicators as inputs to quantitatively predict intervention effects. Results show a classification accuracy of 86.2%, precision of 84.7%, F1 score of 85.4%, and AUC-ROC value of 0.875. The lower limits of the 95% confidence intervals for all indicators are above 0.8, indicating that the model possesses stable and reliable predictive capabilities. Copula entropy effectively captures the nonlinear relationship between self-efficacy and mental health indicators, overcoming the limitations of traditional linear models. The Naive Bayes algorithm reduces the computational complexity of high-dimensional psychological data; the fusion of these two methods achieves accurate modeling of complex psychological variable relationships. These results provide the first empirical evidence of the model’s performance, confirming that the theoretical model can be effectively applied to predicting the effects of dance therapy and providing technical support for the development of personalized psychological intervention programs.

## 4 Discussion

This study confirms that dance therapy exerts a positive and consistent effect on improving self-efficacy among college students. As a mind–body intervention, dance therapy integrates physical movement, emotional expression, and social interaction into a unified process, which differs fundamentally from conventional physical exercise that focuses merely on physical fitness. Through structured movement practice, participants gradually gain a sense of control over their bodies and emotions, which in turn enhances their perceived competence and confidence. Such mastery experience is a key driver of self-efficacy development. Additionally, the group-based nature of dance therapy fosters interpersonal communication, emotional sharing, and mutual support, which help reduce feelings of isolation and self-doubt. These social experiences further consolidate self-efficacy and promote adaptive psychological functioning. The present findings support the notion that mind–body interventions can serve as effective non-pharmaceutical approaches to strengthen psychological resources, especially for young adults facing increasing academic and social pressure.

The results reveal that dance therapy significantly alleviates a wide range of psychological symptoms and contributes to overall mental health improvement. The mechanisms behind this effect are multifaceted. First, physical movement in dance therapy helps release accumulated psychological tension and somatic discomfort, which are common manifestations of stress and anxiety among college students. Rhythmic and expressive movements can regulate autonomic nervous system activity, promoting relaxation and emotional balance. Second, dance therapy provides a nonverbal channel for emotional release, allowing participants to express inner feelings that may be difficult to articulate. This emotional catharsis reduces psychological repression and mitigates symptoms such as anxiety and interpersonal sensitivity. Third, the mindfulness elements embedded in dance therapy encourage present-moment awareness, which helps students disengage from rumination and negative thinking patterns. Collectively, these mechanisms enable dance therapy to address psychological distress from both physical and emotional dimensions, making it a holistic and effective intervention for college mental health.

From a practical perspective, this study highlights the potential of dance therapy as an accessible, low-cost, and sustainable intervention that can be integrated into campus mental health services. It offers a new pathway for universities to diversify their psychological support systems and promote proactive mental health prevention. Despite these contributions, several limitations should be acknowledged. First, the intervention duration is relatively limited, and long-term follow-up data are lacking, so the sustainability of the effects remains to be verified. Second, this study focuses on general psychological outcomes, and more nuanced analyses of specific psychological constructs are needed in future research. Third, the sample is drawn from a single university, which may limit the generalizability of the findings. Future studies could adopt multi-center designs, extend the intervention period, and combine qualitative methods to further explore the underlying mechanisms and optimize intervention protocols.

## 5 Conclusion

The college period is a very critical stage in the process of personal development. A total of 500 college students were initially recruited for this study, with 462 valid participants included in the final statistical analysis. All valid participants were equivalent in terms of physical fitness at baseline. The main difference between the two tests is that the time and energy invested in the movement was significantly higher than before participating in the regular and systematic dance movement training in spare time. In this study, by comparing and analyzing the SCL-90 test results of college students before and after participating in dance training for 3 months, we can see that this sport can not only shape the body and improve the physical quality of college students, but also make the dance movement and aesthetic education have a better combination. Dance can improve the mental health of college students precisely because of the characteristics of the exercise in the training process and the purpose of the exercise. Therefore, we can basically attribute the difference between the two groups’ mental health levels before and after training to dance therapy. From this, it can be speculated that the effect of fitness and dance therapy training on the mental health of college students is positive, effective, obvious and direct.

## Data Availability

The original contributions presented in the study are included in the article/supplementary material, further inquiries can be directed to the corresponding author.
